# Iterative Intraperitoneal Chemotherapy in Gastric Cancer Peritoneal Carcinomatosis

**DOI:** 10.3390/cancers17020289

**Published:** 2025-01-17

**Authors:** Fatemeh Tajik, Belain Eyob, Aaqil M. Khan, Vinodh Kumar Radhakrishnan, Maheswari Senthil

**Affiliations:** 1 Department of Surgery, University of California Irvine Medical Center, Orange, CA 92868, USA; ftajik@hs.uci.edu (F.T.); aaqilk1@hs.uci.edu (A.M.K.); vinodhkr@hs.uci.edu (V.K.R.); 2 Division of Surgical Oncology, Department of Surgery, University of California Irvine Medical Center, Orange, CA 92868, USA; belaine@hs.uci.edu

**Keywords:** gastric cancer, peritoneal carcinomatosis, intraperitoneal (IP) chemotherapy, heated intraperitoneal chemotherapy (HIPEC), normothermic intraperitoneal chemotherapy (NIPEC), pressurized intraperitoneal aerosolized chemotherapy (PIPAC), cytoreductive surgery (CRS)

## Abstract

**Background/objectives:** Despite the incremental improvement of survival with systemic therapy in metastatic gastric cancer (GC), the outcomes of patients with peritoneal carcinomatosis (PC) remain poor. The limited effectiveness of systemic therapy is attributed to the blood–peritoneal barrier and anarchic intra-tumoral circulation, which reduce the penetration of systemic therapy. Approaches that incorporate intraperitoneal (IP) chemotherapy, in addition to systemic therapies, may be a viable alternate strategy. Therefore, we provide a review of biology of gastric cancer peritoneal metastasis and evidence for bidirectional iterative IP chemotherapy in GCPC. **Methods:** A comprehensive search in PubMed, Scopus, Embase, Web of Science, Google Scholar, and ClinicalTrials.gov was performed to find the relevant articles and ongoing phase II/III clinical trials in iterative IP chemotherapy in GCPC. **Results:** Intraperitoneal (IP) chemotherapy leverages the blood–peritoneal barrier to allow for the administration of high concentrations of chemotherapy directly to the peritoneal metastases, with a significant reduction in the systemic toxicity and enhanced drug efficacy against peritoneal metastasis. This pharmacokinetic advantage of IP chemotherapy can be further enhanced by additional measures such as heat or aerosolization. There are three IP chemotherapy approaches, namely, heated intraperitoneal chemotherapy (HIPEC), pressurized intraperitoneal aerosolized chemotherapy (PIPAC), and normothermic intraperitoneal chemotherapy (NIPEC). Recent evidence suggests that iterative IP chemotherapy combined with systemic therapy may offer significant survival benefits for patients with peritoneal metastasis. Furthermore, bidirectional treatment approaches may also increase the chances of surgical resection and survival. **Conclusions:** IP chemotherapy plays a pivotal role in the management of gastric carcinomatosis, particularly in combination with cytoreduction in highly selected patients. The combination of systemic and regional control may increase the chances of surgical resection and may ultimately lead to significant survival benefits.

## Simple Summary

Gastric cancer patients with peritoneal carcinomatosis (PC) face poor outcomes primarily due to the limited efficacy of systemic therapy, which is impeded by the blood–peritoneal barrier and disorganized intra-tumoral circulation. Intraperitoneal (IP) chemotherapy offers a solution by delivering high concentrations of chemotherapy directly to peritoneal metastases, resulting in enhanced efficacy and reduced systemic toxicity. There are three IP chemotherapy approaches, namely, heated intraperitoneal chemotherapy (HIPEC), pressurized intraperitoneal aerosolized chemotherapy (PIPAC), and normothermic intraperitoneal chemotherapy (NIPEC). Combining iterative IP chemotherapy with systemic therapy shows promise for improving survival and increasing surgical resection rates. This review explores the biology of gastric cancer peritoneal metastases and the benefits of bidirectional iterative IP chemotherapy.

## 1. Introduction

Gastric cancer (GC) is the fifth most prevalent cancer and the fifth leading cause of cancer-related deaths globally in 2022 [1]. In the United States, it has been estimated that approximately 26,890 new cases of GC and 10,880 deaths will occur in 2024 [2]. The poor 5-year survival rate of 31% is attributed to late stages at diagnosis and high incidence of metastasis [3,4,5]. The peritoneal cavity is a common site of both synchronous and metachronous metastasis [5,6,7]. The incidence of synchronous peritoneal carcinomatosis (PC) in GC ranges from 10–40%, with a median overall survival (OS) of 5–9 months, which is significantly lower than the survival of other metastatic sites [8,9,10].

Management of metastatic gastric cancer has significantly evolved with the introduction of biomarker-based targeted therapies based on microsatellite status, HER2 amplification, PD-L1 CPS score, and more recently, Claudin 18.2 expression. Several recent phase III clinical trials in first-line treatment of metastatic gastric cancer have unequivocally shown an OS benefit with the addition of targeted agents or immune checkpoint inhibitors to systemic therapy compared to systemic therapy alone. In the KEYNOTE-859 study, a multicenter phase 3 randomized clinical trial that studied the benefit of the addition of pembrolizumab to systemic chemotherapy revealed a significantly improved median OS in the pembrolizumab plus systemic therapy group compared to systemic chemotherapy alone across all populations (intention-to-treat (ITT) population: 12.9 vs. 11.5 months; PDL-1 combined positive score (CPS) ≥ 1 participants: 13.0 vs. 11.4 months; PDL-1 CPS ≥ 10 participants: 15.7 vs. 11.8 months) [11]. Similarly, in the updated 4-year analysis of the CHECKMATE 649 trial, combination therapy of Nivolumab with mFOLFOX6 chemotherapy showed significant improvement in OS compared to the chemotherapy group in all randomized patients (13.7 vs. 11.6 months) [12,13,14].

More recently, two separate phase III clinical trials, SPOTLIGHT and GLOW, have shown that Zolbetuximab, a monoclonal antibody to Claudin 18.2, when combined with systemic therapy, resulted in improved median OS compared to systemic therapy alone (SPOTLIGHT: 18.23 vs. 15.54 months, HR = 0.750, 95% CI: 0.601-0.936; GLOW: 14.39  vs. 12.16 months, HR = 0.771, 95% CI: 0.615–0.965) [15,16]. Although these trials have shown survival benefits in metastatic gastric cancer, the benefits of these therapies are less promising in the subset of gastric cancers that have a high predilection to metastasize to the peritoneum, namely, diffuse type, poorly differentiated, and signet ring histologies In the GLOW study, the combination of Zolbetuximab with CAPEOX compared to CAPEOX alone showed a lower OS benefit in the diffuse type (14.32 vs. 12.55 months, HR = 0.726, 95% CI: 0.46–1.06) as compared to the intestinal type (17.84 vs. 12.7 months, HR = 0.702, 95% CI: 0.40–1.22) [15]. Similarly, in the 3-year follow up of the Checkmate 649 study, even in the PD-L1 CPS > 5 subgroup, the survival in the signet ring group (12.1 vs. 10.1 months, HR = 0.67, 95% CI: 0.47–0.96) was less robust compared to the non-signet ring group (15.0–12.3 months, HR = 0.69, 95% CI: 0.59–0.80) [13,14].

Despite the incremental impact of systemic therapy on peritoneal metastases, there is a significant need for alternate treatment strategies to improve survival. It has been established that the limited effectiveness of systemic chemotherapy is mostly due to the blood–peritoneal barrier and anarchic intra-tumoral circulation, which prevent the penetration of systemic therapy in PC [17,18,19]. Hence, approaches that incorporate intraperitoneal (IP) chemotherapy, in addition to systemic therapies, may be a viable alternate strategy. This bidirectional approach could potentially be combined with cytoreduction in appropriately selected patients to improve the survival outcomes of GCPC patients. The main advantage of IP treatment is the ability to deliver high concentrations of chemotherapeutic drugs directly to peritoneal metastases, allowing for more targeted treatment of the disease. This regional approach significantly reduces the systemic toxicity associated with traditional chemotherapy, while enhancing the drug’s efficacy against cancer cells in the peritoneal cavity [20,21]. Additionally, there may be a synergistic effect between systemic and IP therapy that could lead to better disease control.

In this review, we provide an overview of the biology of PC in gastric cancer and discuss the rationale for IP chemotherapy in the management of GCPC. We also compare the different types of iterative IP therapies, namely, heated intraperitoneal chemotherapy (HIPEC), pressurized intraperitoneal aerosolized chemotherapy (PIPAC), and normothermic intraperitoneal chemotherapy (NIPEC), and the ongoing phase II/III clinical trials.

## 2. Biology of Peritoneal Metastases

The peritoneum consists of a single layer of mesothelial cells adherent to a basement membrane that covers the sub-mesothelial stroma. This structure, along with other components such as stromal cells, interstitial cells, and the capillary wall, make the blood–peritoneal barrier, forming a mechanical barrier for the diffusion of systemically administered chemotherapeutic agents into the peritoneal cavity [22,23,24].

**Figure 1:**
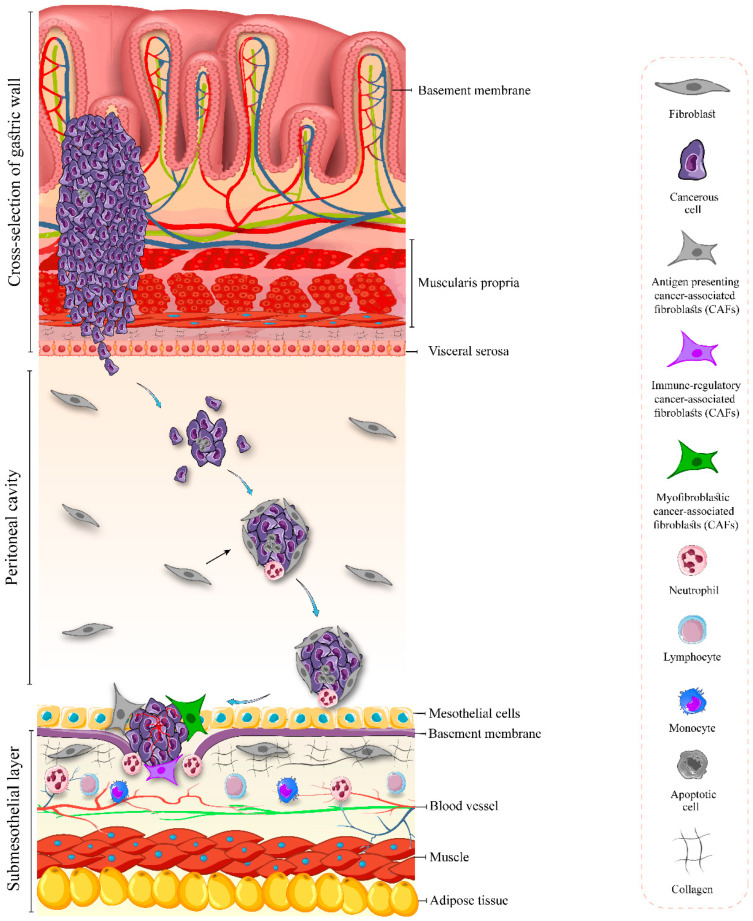
The sequence of events leading to peritoneal dissemination in gastric cancer. This process consists of five main stages: detachment of single cancer cells or clumps from the primary tumor through the serosa; acclimatization and survival within the peritoneal cavity microenvironment and recruitment of fibroblasts; attachment to the mesothelial layer; invasion into the submesothelial area; and proliferation of metastatic nodules.

The principle concept of tumor metastasis, defined as the “seed and soil” theory by Stephen Paget, illustrates the crosstalk between the cancer cells and the local microenvironment of the metastatic sites [25]. According to this theory, peritoneal dissemination in gastric cancer is described as the bidirectional crosstalk between gastric cancer cells and mesothelium through interactions between extracellular matrix proteins and tumor cell adhesion molecules [26]. This process consists of five main stages: detachment of single cancer cells or clumps from the primary tumor through the serosa; acclimatization and survival within the peritoneal cavity microenvironment and recruitment of fibroblasts; attachment to the mesothelial layer; invasion into the submesothelial area; and proliferation as metastatic nodules within the peritoneum [27,28,29,30]. The peritoneal tumor nodules might have vasculature; however, the vasculature supporting peritoneal metastases is often anarchic and poorly organized and may not be completely cohesive, resulting in inefficient blood supply [18]. This disorganized vasculature, along with the presence of the blood–peritoneal barrier, limits the efficacy of systemic chemotherapy due to inadequate drug penetration into the peritoneal cavity. IP chemotherapy presents a viable alternative by allowing direct contact between chemotherapeutic agents and peritoneal metastases (Figure 1).

## 3. Pharmacokinetics of Intraperitoneal Chemotherapy

The utilization of IP chemotherapy provides several pharmacokinetic advantages compared to systemic therapy alone. Medications administered directly within the peritoneal cavity have the benefit of being retained in the peritoneal cavity for longer periods of time due to the blood–peritoneal barrier and provide a higher cytotoxic dose than the systemic route of administration [21]. The blood–peritoneal barrier acts as a selective filter, permitting the passage of water and small solutes but limiting the entry of large molecules based on their size, charge, or hydrophobicity [17,31,32]. Hence, medications with high molecular weight and hydrophobicity have long retention times and may be best suited for IP administration [33]. Paclitaxel (PTX) is often the agent of choice for normothermic intraperitoneal treatment in GCPC due to the aforementioned pharmacokinetic advantages [34,35]. Administration of chemotherapy intraperitoneally not only leverages the blood–peritoneal barrier for retaining high concentrations of the drug intraperitoneally but also takes advantage of the circulation of peritoneal fluid to aid in the wide distribution and contact of chemotherapy with the large surface area of the peritoneum. Since the mechanism of action of IP chemotherapy agents is by diffusion through the surface of the metastatic nodules, iterative administration of IP chemotherapy is necessary for adequate disease control.

## 4. Rationale for Bidirectional Chemotherapy

The failure of recent randomized control trials that evaluated the role of cytoreductive surgery (CRS) with HIPEC after systemic therapy alone highlights the importance of intraperitoneal disease control prior to cytoreduction. GASTRIPEC-I, a German multicenter phase III clinical trial, sought to compare the survival benefit of CRS/HIPEC in comparison to CRS [36]. Out of 105 patients enrolled, 55 patients had progression of disease on neoadjuvant chemotherapy or died, making them ineligible for surgery. Nevertheless, patients who underwent CRS/HIPEC had 7.1 months of progression-free survival (PFS) as compared to 3.5 months in the CRS-only group. Despite improvement in PFS, the OS was similar between the two groups (14 months). Furthermore, only 47.4% of patients were able to achieve complete cytoreduction at the time of surgery. In the recently reported RENAISSANCE FLOT 5 trial, which evaluated the role of surgery after systemic therapy in patients with limited metastatic disease, patients with peritoneal disease who underwent surgery had the worst OS (12 vs. 19 months), and in fact, surgery was deemed detrimental in this group [37,38]. Data from previous CRS studies are also similarly discouraging, with a PFS of less than 12 months and a 50% recurrence rate isolated to the peritoneum [39]. The recognition and acknowledgment of the shortcomings of systemic therapy alone and the likelihood of synergism between systemic and intraperitoneal therapy have influenced the incorporation of IP chemotherapy with systemic therapy. Delivery of IP chemotherapy in conjunction with systemic therapy has demonstrated both safety and efficacy in other peritoneal surface malignancies [40,41]. Contemporary studies exploring the use of bidirectional therapy in GCPC highlight several potential gaps in the current therapeutic approach that bidirectional therapy may be able to address. Introducing regional IP therapies concomitantly with systemic therapy for advanced GC could augment the response of both the primary tumor as well as macroscopic tumor deposits in the peritoneum. This has potential implications for prolonging disease-free survival, reducing complications such as malignant obstructions and ascites that are associated with worse quality of life (QoL) in advanced stages, as well as converting a subset of patients with limited peritoneal disease into potential surgical candidates.

## 5. Intraperitoneal Chemotherapy Approaches

The three main approaches for IP therapy are heated intraperitoneal chemotherapy (HIPEC), normothermic intraperitoneal chemotherapy (NIPEC), and pressurized intraperitoneal aerosolized chemotherapy (PIPAC). Each of these approaches has unique pharmacokinetic advantages and tumor microenvironment changes that facilitate treatment response. A comparison of the three approaches pertaining to expertise, hospital stay, and cost is shown in Table 1.

### 5.1. Iterative Heated Intraperitoneal Chemotherapy (HIPEC)

Although HIPEC is typically used in conjunction with CRS, there are a limited number of studies that have explored the use of iterative HIPEC. Badgwell et al. conducted a phase II single-arm clinical trial to assess the safety, feasibility, and efficacy of iterative laparoscopic HIPEC in GCPC [42]. Nineteen patients with either cytology-positive GC or image occult PC in the absence of other solid organ or visceral metastasis were enrolled in this study. Patients had received a median of 8 cycles of systemic chemotherapy prior to enrollment. Laparoscopic HIPEC with mitomycin c (30 mg) and cisplatin (200 mg) for 60 min was administered every three weeks for a maximum of 5 cycles. Patients who had no evidence of carcinomatosis or conversion to negative cytology were eligible for gastrectomy. Seven out of nineteen patients had a complete response, out of which five had gastrectomy. Of the five patients who underwent gastrectomy, four had only cytology-positive disease. Median OS for the entire cohort was 20.3 months, and for the gastrectomy group, the time of resection was 29 months. There were no 30-day mortalities, and procedure-related complications were low (11%). Following this initial study, Badgwell et al. conducted another single-arm phase II study with a slightly different approach in which patients with cytology positive or GCPC who had completed systemic therapy and at least one laparoscopic HIPEC were enrolled to undergo cytoreduction and HIPEC. Of the 20 patients in the study, 15 had a single laparoscopic HIPEC and 5 had two laparoscopic HIPEC. The median PCI was 2 (range of 0–13). The median OS from the diagnosis of metastatic disease was 24.2 months and from the time of CRS and HIPEC was 16.1 months [43]. The difference in the median survival from the surgical resection between the two studies (29 months vs. 16.1 months) can possibly be attributed to the number of HIPEC procedures and the burden of disease. In the 2017 study, patients who underwent gastrectomy received 5 cycles of iterative HIPEC compared to the 2021 study in which 75% of the patients received only one HIPEC procedure prior to cytoreduction. Secondly, in the first study, 4/5 patients had only cytology-positive disease in contrast to the second study, in which 12/20 patients had gross PC. Although these two studies cannot be directly compared, the survival differences indicate that iterative HIPEC may provide better disease control compared to a single exposure.

#### Ongoing Phase II/III HIPEC Trials

There are three ongoing phase II or III clinical trials currently designed to evaluate the efficacy and safety of neoadjuvant HIPEC in combination with systemic chemotherapy, with outcomes focused on survival, resection rates, and adverse events (Table 2).

Cui et al. is conducting a multicenter, randomized phase III trial in China, evaluating the efficacy of three cycles of neoadjuvant HIPEC using three separate drugs successively: IP PTX, cisplatin, and raltitrexed combined with systemic chemotherapy (S-1) in GCPC patients with PCI < 20 [44]. After the neoadjuvant iterative HIPEC, participants in the experimental group will undergo CRS followed by three additional successive cycles of HIPEC with the three drugs and 4–6 cycles of adjuvant systemic chemotherapy, while patients in the control group receive systemic chemotherapy (S-1) alone, followed by CRS and adjuvant systemic treatment. Another multicenter, randomized phase III trial in China (NCT05228743) is investigating the benefit of adding iterative HIPEC with IP PTX (at least four times), followed by bidirectional systemic therapy with IP and IV PTX after three to six weeks of HIPEC completion in GC patients with limited peritoneal metastasis (stage ≤ P1b). The control group will receive bidirectional therapy without the initial 4 cycles of HIPEC. After 2–4 cycles of bidirectional treatment, a restaging evaluation is done to assess treatment response in both groups. Patients with stages P0 or P1a are deemed candidates for open radical gastrectomy (D2/D2 +). Patients with P1b/c cases will continue the original treatment until disease progression [45]. There is an ongoing phase II nonrandomized clinical trial, in which patients with limited PC (stage P1a or P1b) receive one cycle of HIPEC (IP PTX) with systemic chemotherapy (S-1), followed by three additional cycles of systemic chemotherapy, CRS with HIPEC, and adjuvant systemic treatment. However, patients with extensive PC (stage P1c) undergo a similar regimen with an additional cycle of HIPEC and systemic treatment without any surgery [46]. These trials aim to determine whether incorporating preoperative HIPEC into treatment regimens combined with CRS and HIPEC in selected patients with limited disease improves survival. However, iterative HIPEC does have limitations as it requires repeated exposure to general anesthesia and a brief interruption in systemic chemotherapy that is crucial in mitigating systemic disease spread.

### 5.2. Iterative Pressurized Intraperitoneal Aerosolized Chemotherapy (PIPAC)

In contrast to HIPEC, the cytotoxic agents in PIPAC are nebulized at high pressure with a micropump and delivered at normothermia into the abdominal cavity after attaining pneumoperitoneum. This technology was largely developed to address locoregional tumor control in advanced gastric cancer patients with high PCI in whom surgical resection was not possible. The pressurized aerosol was thought to maintain a more homogeneous distribution compared to HIPEC, with the high pressures also overcoming the tumor interstitial barrier, thus achieving a higher therapeutic concentration as compared to HIPEC [47]. This unique pharmacokinetic property translated into a clinical pharmacodynamic advantage, with research showing improved control of GCPC as well as demonstrating benefits in OS [48]. The application of PIPAC in bidirectional therapy may have potential benefits in achieving better tumor control in GCPC. A research group in Russia supported the above findings in their phase II single-arm clinical trial, where they explored the added benefit of bidirectional PIPAC every 6 weeks with systemic chemotherapy in 31 patients with GCPC. They found that the procedure was well tolerated without any major complications. Though 26% had evidence of disease progression, 9 out of 15 patients who had iterative procedures showed evidence of significant disease response, of which 4 had a complete pathologic response [49]. These promising results have encouraged several randomized controlled trials to further investigate the feasibility of this approach in bidirectional therapy, which are currently underway.

#### Ongoing Phase II/III PIPAC Trials

The ongoing phase II and III PIPAC trials for GCPC are listed in Table 3. The VerONE trial, a multicenter, randomized phase III study, was designed to assess the efficacy of PIPAC treatment combined with systemic chemotherapy in patients with limited peritoneal disease (cytology+ and/or PCI ≤ 6) [50,51]. Patients in the experimental group will receive bidirectional treatment such as PIPAC (IP cisplatin and doxorubicin) and the FOLFOX regimen. Patients with disease progression will transition to second-line therapy, while those with stable disease or a positive treatment response who are surgical candidates will undergo CRS. Similar to this trial, Luksta et al. also initiated a phase II clinical trial evaluating the efficacy and treatment response of PIPAC (cisplatin and doxorubicin) in conjunction with FOLFOX in GC patients with peritoneal metastasis, regardless of their PCI score [52,53]. The SPECTRA phase II clinical trial focuses on assessing the safety and feasibility of PIPAC in GCPC patients with minimal peritoneal disease (cytology+ or PCI ≤ 3) [54]. Participants will undergo three cycles of PIPAC (IP cisplatin and doxorubicin), alternating with standard systemic chemotherapy. Patients achieving negative peritoneal cytology and a PCI of 0 during restaging laparoscopic assessment will be considered for gastrectomy with D2 lymphadenectomy surgery. These trials aim to provide insights into the benefits of bidirectional treatment of PIPAC and systemic chemotherapy in terms of resectability, survival, recurrence, and quality of life for patients with limited peritoneal carcinomatosis.

### 5.3. Iterative Normothermic Intraperitoneal Chemotherapy (NIPEC)

NIPEC remains the most studied intraperitoneal approach due to its pharmacokinetic and logistical advantages. As described earlier, the unique pharmacokinetics of taxanes allow for the administration of high doses of drugs with limited systemic absorption and low systemic toxicity [55]. NIPEC can be administered without interruption of systemic therapy. Furthermore, delivery via a subcutaneous port eliminates the need for repeated exposure to surgical procedures under general anesthesia and the need for hospital admission, all the while providing an avenue for the therapeutic drainage of symptomatic ascites during treatment if necessary. These potential added benefits to patients’ QoL, however, are yet to be fully explored, nor are they reported in the existing literature. PHOENIX GC, the largest phase III randomized controlled trial from Japan to date, compared the survival benefit of IP PTX with IV PTX and S-1 vs. treatment with just systemic chemotherapy (cisplatin and S1) for GCPC in 183 patients [56]. Though they were not able to demonstrate difference in OS between the two groups, 3-year survival in the experimental group was 21.9% vs. 6% in the control arm. To further investigate the potential benefits of bidirectional IP PTX, Lin et al. from China conducted the FNF-004 trial: a phase II randomized clinical trial evaluating differences between three groups (mFOLFOX6 alone vs. IV PTX plus mFOLFOX6 vs. IP PTX plus mFOLFOX6) in metastatic GC [57]. They showed that both IV and IP PTX in addition to mFOLFOX6 were superior to mFOLFOX6 alone, with median PFS of 6.52, 5.83, and 4.55 months, respectively. Though they were not able to demonstrate difference between IV and IP PTX, the addition of IP was both feasible and well tolerated. Two recent phase II trials from Singapore and Japan have reported that the addition of IP PTX to systemic chemotherapy is feasible and is associated with better PFS and OS [58,59]. In the study by Chia et al., patients with gastric PC *(n* = 44) received IP PTX (40 mg/m^2^) on days 1 and 8 of three-week cycles combined with CAPEOX for a total of eight cycles [58]. They utilized a combination of cross-sectional imaging and cytologic evaluation to determine whether patients responded to treatment or confirm the absence of disease progression. Compared to historical control patients who received systemic chemotherapy alone, both PFS (9.5 vs. 4.4 months HR—0.39 (CI 0.25–0.66) and OS (14.6 vs. 10.6 months HR—0.44 (CI 0.26–0.74)) were significantly better in the IP PTX group. Furthermore, those who were deemed fit for conversion surgery demonstrated an even higher median OS of 29.9 months. Minor port-related complications such as superficial infections and leakage requiring conservative management were reported for 10/44 in the experimental group, whereas 9% (4/44) required a second procedure for port adjustment. Similarly, in a phase II trial, Kobayashi et al. were able to demonstrate a median OS of 19.4 months (6.1–24.6 months) and PFS 11.1 months (8.4–15.9 months) when adding 20 mg/m^2^ of IP PTX to the Japanese standard of care systemic chemotherapy of oral S1 and IV cisplatin (*n* = 53) [59]. The cumulative advantage of surgical resection with curative intent in carefully selected patients was again evident following IP PTX. The subgroup analysis in the 16/53 patients who underwent gastrectomy had both improved median survival time and PFS (42.1 months and 18.1 months, respectively). Port-related complications were minor and occurred in 4/53 patients.

#### Ongoing Phase II/III NIPEC Trials

Bidirectional NIPEC treatment combined with systemic chemotherapy has been extensively evaluated in several clinical trials due to its feasibility and cost-effectiveness. Hence, we summarized the ongoing phase II/III clinical trials in China, USA, and Brazil (Table 4). The DRAGON-01 study, a multicenter randomized controlled phase III trial, is investigating the efficacy of NIPEC with systemic chemotherapy in GCPC patients [60]. The experimental group will receive the neoadjuvant NIPEC (IP PTX) with IV PTX plus oral S-1 (NIPS), whereas the control group will receive only a systemic chemotherapy regimen. During the restaging laparoscopy, if a response is observed, a gastrectomy with D2 lymphadenectomy will be performed, followed by adjuvant NIPS. The DRAGON-01 trial has been completed, and the results are scheduled to be presented at the ASCO GI conference in 2025.

The STOPGAP trial is a single-center, phase II trial assessing the efficacy of iterative NIPEC (IP PTX) in conjunction with systemic therapy (IV PTX, 5-FU, and leucovorin) in gastric and GEJ adenocarcinoma patients with either cytology + or PC with no extraperitoneal disease following systemic therapy for a minimum of three months. The systemic regimen is as determined by their oncologist based on tissue biomarkers [61,62]. Patients undergo bidirectional therapy with NIPEC PTX 40 mg/m^2^ on days 1 and 8. Targeted therapies are allowed along with systemic treatment. Restaging imaging and laparoscopic evaluation are done after four cycles. Patients who have a response and a PCI ≤ 10 may undergo CRS if complete cytoreduction is feasible, and if PCI > 10, IP treatment will be continued beyond four cycles. Critically, the trial also aims to assess patient reported QoL via the EuroQol- 5 dimensions-5 level (EuroQol-5D-5L) questionnaire, which will be completed every 8 weeks, addressing a gap in the current NIPEC literature on QoL. Results from STOPGAP-I have already informed the approval of STOPGAP II, EA 2234, a phase II/III, randomized, multicenter clinical trial of bidirectional PTX in GCPC, which will begin patient enrollment in early 2025. Dias et al. have initiated a single-center phase II trial evaluating the safety, tolerability, and peritoneal response rate of IP PTX with systemic chemotherapy in GC patients with cytology + or PC (PCI ≤ 12) [63,64]. After four cycles, patients with clinical and radiographic peritoneal response will undergo restaging laparoscopy, and if a complete response is observed, will be considered a candidate for gastrectomy. There is another single-arm phase II ongoing trial being conducted to investigate the efficacy of bidirectional chemotherapy using IP and IV PTX and oral nilotinib in PC patients with gastrointestinal and gynecology primary tumors [65]. The primary aim of this study is to calculate the rate of downstaging of peritoneal disease burden to become resectable based on PCI.

These ongoing trials highlight the potential of bidirectional NIPEC combined with systemic chemotherapy as a promising approach for GCPC treatment.

## 6. Role of Cytoreductive Surgery After IP Chemotherapy

With the body of evidence described thus far supporting the potential for regional and systemic control of GCPC with bidirectional therapy, research is beginning to explore the prospects of implementing neoadjuvant bidirectional therapy to convert patients with PC-only metastatic disease into surgical candidates. Since the majority of patients will continue to have active disease in the primary site despite excellent response in the peritoneum, the role of gastrectomy or cytoreduction should be explored in a systematic fashion. The existing data suggests that bidirectional therapy can improve macroscopic control of peritoneal disease, ultimately allowing for more patients with advanced GC to be considered for surgery, with the best chance for complete cytoreduction. Discerning which patient population with GCPC should undergo CRS cannot be overemphasized [66]. The extent of peritoneal disease burden, response to the treatment of peritoneal disease, and the ability to achieve complete cytoreduction are all factors that bear significance in the selection of patients for CRS. The other aspect of treatment that will have an impact on survival is the adjuvant treatment post CRS. These remain areas that need further investigation.

The results of the DRAGON-01 trial, which are soon to be released, will be extremely helpful in providing insights into patient selection for gastrectomy and the optimal post-surgical treatment [60].

## 7. Conclusions and Future Directions

Intraperitoneal chemotherapy plays a pivotal role in the management of gastric carcinomatosis, particularly in combination with cytoreduction in highly selected patients. There are some key factors for effective IP chemotherapy delivery, including the selection of the right drug, the right conditions, duration of exposure, and synergism with systemic therapy. The combination of systemic and regional control may increase the chances of surgical resection and may ultimately lead to significant survival benefits. The ongoing trials will hopefully elucidate the impact of bidirectional therapy in GCPC and the addition of surgical treatment in appropriately selected patients, ultimately moving the needle forward and shifting the current treatment paradigm in GCPC.

## Figures and Tables

**Table 1:** Comparison of intraperitoneal chemotherapy including HIPEC, PIPAC, and NIPEC.

HIPEC Heated Intraperitoneal Chemotherapy	PIPAC Pressurized Intraperitoneal Aerosolized Chemotherapy	NIPEC Normothermic Intraperitoneal Chemotherapy
Administered in the operating room	Administered in the operating room	Administered in outpatient setting
Requires hospital stay	Requires hospital stay	No hospital stay
Requires special expertise	Requires special expertise	No special expertise
High cost	High cost	Low cost

**Table 2:** Ongoing phase II/III clinical trials of iterative heated intraperitoneal chemotherapy (HIPEC) combined with systemic chemotherapy in gastric cancer peritoneal carcinomatosis.

Author, Registration Number	Country	Year	Study Design	Patient No.	Study Arms		Outcomes/Endpoints	
					Control	Experimental	Primary	Secondary
Cui, et al., [44] NCT03179579	China	2017–2022	Multicenter phase III, randomized	88	SC (S-1) 3 cycles followed by: CRS + adjuvant SC 4–6 cycles	HIPEC (PTX/cisplatin/raltitrexed) 3 times + SC (oral S-1) 2 cycles followed by: CRS + HIPEC 3 times + adjuvant SC 4–6 cycles	3-yrs OS	Risk factors for morbidity and mortality
NCT05228743 [45]	China	2020–2023	Multicenter phase III, randomized	180	Bidirectional therapy (IV PTX + IP PTX + oral S-1)	HIPEC (PTX) at least 4 times, after 3–6 weeks bidirectional therapy (IV PTX + IP PTX + oral S-1)	R0 resection rate	1-yr OS
Wang, et al., [46] NCT05095467	China	2021–2026	Single center phase II, non-randomized	157	NA	Limited PC (stage P1a or P1b): 1 cycle HIPEC (IP PTX) + SC (S-1), sequential 3 cycles SC, surgery + HIPEC, adjuvant SC Extensive PC (stage P1c): 1 cycle HIPEC (IP PTX) + SC (S-1), sequential 3 cycles SC, HIPEC (IP PTX) + SC (S-1), sequential 3 cycles SC	3-yrs OS	3-yrs PFS, adverse events

HIPEC, heated intraperitoneal chemotherapy; IP, intraperitoneal; IV, intravenous; CRS, cytoreductive surgery; SC, systemic chemotherapy; yr, year; PTX, paclitaxel; OS, overall survival; PFS, progression-free survival; PC, peritoneal carcinomatosis; NA, not applicable.

**Table 3:** Ongoing phase II/III clinical trials of iterative pressurized intraperitoneal aerosolized chemotherapy (PIPAC) combined with systemic chemotherapy in gastric cancer peritoneal carcinomatosis.

Author, Registration Number	Country	Year	Study Design	Patient No.	Study Arms		Outcomes/Endpoints	
					Control	Experiment	Primary	Secondary
PIPAC VerONE Casella et al., [50,51] NCT05303714	Italy	2022–2028	Multicenter phase III, 1:1 randomization	98	SC (FOLFOX)	SC (FOLFOX) + PIPAC (cisplatin/doxorubicin) every 2 chemotherapy cycles	Resectability rate	OS, PFS, disease-related survival, peritoneal regression grade score, tumor regression grading, QoL, complication rate
Luksta et al., [52,53] NCT05644249	Lithuania	2022–2027	Multicenter phase II, nonrandomized	37	NA	PIPAC (cisplatin/doxorubicin) every 2 chemotherapy cycles + SC (FOLFOX) × 6 cycles	Objective tumor response rate 1 week after second PIPAC	Compliance to treatment, postoperative complication, PCI and histological regression, ascites volume, tumor marker levels, QoL, 2-yrs OS, PFS, adverse events
SPECTRA Hanna et al., [54] NCT05318794	United Kingdom	2025–2030	Single center phase II, nonrandomized	20	NA	PIPAC (cisplatin/doxorubicin) + standard SC	Feasibility and safety	Tumor regression, patient morbidity, QoL, 5-yr disease recurrence, OS

PIPAC, pressurized intraperitoneal aerosolized chemotherapy; SC, systemic chemotherapy; yr, year; OS, overall survival; PFS, progression-free survival; PCI, peritoneal cancer index; QoL, quality-of-life; FOLFOX, leucovorin/5-fluorouracil/oxaliplatin; NA, not applicable.

**Table 4:** Ongoing phase II/III clinical trials of iterative normothermic intraperitoneal chemotherapy (NIPEC) combined with systemic chemotherapy in gastric cancer peritoneal carcinomatosis.

Author, Registration Number	Country	Year	Study Design	Patient No.	Study Arms		Outcomes/Endpoints	
					Control	Experiment	Primary	Secondary
DRAGON-01 Lu et al. [60] ChiCTR-IIR-16009802	China	2022–2028	Multicenter phase III, 2:1 randomization	238	SC (IV PTX + oral S-1)	IP PTX + SC (IV PTX + oral S-1)	OS	Pathological response rate, gastrectomy radicality rate, PFS, adverse events
STOPGAP Senthil et al. [61,62] NCT04762953	United States	2021–2025	Single center phase II, nonrandomized	35	NA	IP/IV PTX + IV 5-FU + IV leucovorin	1-yr PFS, adverse events	1-yr OS, QoL
Dias et al. [63,64] NCT05541146	Brazil	2022–2025	Single center phase II, nonrandomized	30	NA	IP PTX × 4 cycles + SC	Complete peritoneal response rate	6 months PFS, 5-yrs OS
Blakely, et al. [65] NCT05185947	United States	2022–2025	Single center phase II, nonrandomized	70	NA	IP/IV PTX + oral nilotinib	Efficacy of bidirectional treatment	3 yrs-OS, PFS, peritoneal PFS, safety and tolerability of therapy, QoL, clinicopathologic response to therapy

*IP,* intraperitoneal; IV, intravenous; *SC,* systemic chemotherapy; *PTX,* paclitaxel; *yr,* year; *OS,* overall survival; *PFS,* progression-free survival; *5-FU,* 5-fluorouracil; *QoL,* quality-of-life; *NA,* not applicable.
